# Perceptual Stability of the Lissajous Figure Is Modulated by the Speed of Illusory Rotation

**DOI:** 10.1371/journal.pone.0160772

**Published:** 2016-08-25

**Authors:** Veith A. Weilnhammer, Philipp Sterzer, Guido Hesselmann

**Affiliations:** Visual Perception Laboratory, Department of Psychiatry and Psychotherapy, Campus Charité Mitte, Charité Universitätsmedizin, 10117 Berlin, Germany; Radboud Universiteit, NETHERLANDS

## Abstract

Lissajous figures represent ambiguous structure-from-motion stimuli rotating in depth and have proven to be a versatile tool to explore the cognitive and neural mechanisms underlying bistable perception. They are generated by the intersection of two sinusoids with perpendicular axes and increasing phase-shift whose frequency determines the speed of illusory 3D rotation. Recently, we found that Lissajous figures of higher shifting frequencies elicited longer perceptual phase durations and tentatively proposed a “representational momentum” account. In this study, our aim was twofold. First, we aimed to gather more behavioral evidence related to the perceptual dynamics of the Lissajous figure by simultaneously varying its shifting frequency and size. Using a conventional analysis, we investigated the effects of our experimental manipulations on transition probability (i.e., the probability that the current percept will change at the next critical stimulus configuration). Second, we sought to test the impact of our experimental factors on the occurrence of transitions in bistable perception by means of a Bayesian approach that can be used to directly quantify the impact of contextual cues on perceptual stability. We thereby estimated the implicit prediction of perceptual stability and how it is modulated by experimental manipulations.

## Introduction

In bistable vision, perception alternates between two different interpretations of a constant ambiguous sensory input. Novel theories place bistable perception in the context of predictive coding [1, 2]. Within this framework, perception is explained as an active process of inferring on the most likely causes of sensory input [3], which—according to Bayes’ theorem—can be implemented by combining a prior (representing “beliefs” about the world) and a likelihood (representing new sensory input) to estimate the posterior distribution, based on which perceptual decisions are formed [4].

Along this line of thought, the inherently ambiguous stimulation we receive through our senses (the likelihood) is understood to result in a clear and stable experience of our environment (a perceptual decision based on the posterior distribution) after taking into account what we already know about our world (the prior distribution). This idea implies that bistable vision should not be qualitatively different from normal vision, but represents a special case of it, namely oscillations in perceptual decisions that occur when the environment offers little information that—by means of prior predictions—might help to infer on a stable cause of sensory stimulation. Importantly, such cues adding to the perceptual disambiguation of the continuously ambiguous sensory input might originate from many different sources [5] and might have a differential impact on the construction of a stable percept. In this study, we tried to identify “contextual” cues in the visual environment that influence perceptual stability and quantify their impact on the dynamics of bistable perception.

To this end, we studied the perceptual time courses elicited by ambiguous Lissajous figures [6]. Lissajous figures represent ambiguous motion stimuli which are perceived as objects rotating in-depth and unpredictably changing their direction of rotation. They are generated by 2D curves in the x-y plane obtained by taking x and y to vary sinusoidally; the frequency of the increasing phase-shift of the sinusoids determines the speed of illusory 3D rotation. Even though Lissajous figures are physically ambiguous for all angles of rotation (i.e., all phase offsets between the sinusoids), perceptual transitions almost exclusively occur when the lines composing the stimulus overlap [7], which mirrors the transitions evoked by random-dot-kinematograms displayed in rings [8]. We define self-occlusions (or, overlaps) as the stimulus configurations in which imaginary dots on the Lissajous stimulus can no longer be uniquely associated with either the front or the back of the illusory 3D figure.

In a recent study [9], we investigated the perceptual dynamics of bistable Lissajous figures for two different levels of complexity (i.e., number of cycles per unit space), shifting frequency and line width, and found a significant effect of these factors on perceptual phase durations and transition probabilities:

1. Lissajous figures of higher complexity evoked shorter perceptual phase durations (i.e., higher transition probabilities) when compared to Lissajous figures of lower complexity
2. Lissajous figures of higher shifting frequencies elicited longer perceptual phase durations (i.e., lower transition probabilities) when compared to Lissajous figures at lower shifting frequencies
3. Lissajous figures composed of thinner lines induced longer perceptual phase durations than Lissajous figures composed of thicker lines.

We discussed two possible explanations for these results. On the one hand, transitions occur almost exclusively at self-occlusion events, which are shorter for Lissajous figures of higher shifting frequency and for Lissajous figures that are composed of thinner lines, thus offering a possible explanation for an increase in perceptual stability in these cases. On the other hand, we speculated that the perceptual dynamics of the Lissajous figure might be influenced by the “representational momentum” of the stimulus [10]. In analogy to the rotation of physical objects, the illusory rotational speed would stabilize the (perceived) direction of rotation, as it would afford more “energy” to reverse the direction of rotation of a physical object revolving at a higher speed, and could thus be considered a stabilizing ‘contextual’ cue in the visual environment.

Here, we aimed to disentangle these hypotheses by varying the shifting frequency and size of a Lissajous figure, yielding differential effects on the speed of illusory rotation, the planar speed of the moving sinusoids and the time spent in self-occluding configurations. We thereby tried to identify which factor contributed to modulations in perceptual stability of the Lissajous stimulus. Based on the known behavior of Lissajous parameters (a-e, summarized in Table 1), we formulated the following predictions:

1. If the increased perceptual stability of the Lissajous figure at higher shifting frequency is brought about by the increased speed of illusory rotation (i.e., by the modulation of parameter “e”), the data will show an effect of shifting frequency, but no effect of stimulus size, because the speed of illusory rotation is equal across different sizes of the stimulus. This outcome would further support the notion of “representational momentum”.
2. If the increased perceptual stability of the Lissajous figure at higher shifting frequency is brought about by shorter durations of single self-occlusion events (i.e., by the modulation of “b”), or by the higher planar velocity of the pixels on the screen (i.e., by the modulation of “d”), the data will show similar effects of increased shifting frequency and size, because both these variations lead to a reduced duration of self-occlusion events and to an increased planar velocity. (Note that the duration of single self-occlusion events and planar pixel velocity are inversely related).
3. If total overlap time (i.e., the modulation of “c”) contributes to an increased perceptual stability of the Lissajous figure, one would expect an additional effect of stimulus size (Note that total overlap time does not vary with shifting frequency of the stimulus).

**Table 1:** Experimental Manipulations.

size	shifting frequency	
	slow (0.15 rps)	fast (0.30 rps)
*small*(2.05°)	a	2a
	b	b/2
	c	1c
	d	2d
	e	2e
*big*(4.10°)	1a	2a
	b/2	b/4
	c/2	c/2
	2d	4d
	1e	2e

The influence of the experimental manipulations (factors “shifting frequency” and “size”) on five parameters of the Lissajous figure: (a) the number of self-occlusion events per block; (b) the duration of a single self—occlusion event; (c) the total duration of all self-occlusion events per block (i.e., c = a x b); (d) the planar speed of the sinusoids composing the Lissajous figure; (e) the speed of illusory rotation of the Lissajous figure. The values of parameters a-e are expressed as multiples of the corresponding values for the small, slow Lissajous stimulus

Moving beyond conventional statistical analysis, we also sought to capture the different impact of these factors on the occurrence of transitions in bistable perception by applying a generative predictive coding model of bistable perception that can be used to quantify the impact of contextual cues on predictions of perceptual stability. Thereby, we aimed to describe the inferred implicit stability of the visual display and how it is modulated by our experimental manipulations, framing the perceptual dynamics of a bistable stimulus as subject to prior beliefs. This approach seems particularly apt for investigation of “representational momentum” effects on bistable perception, since the latter have been linked to implicit prior beliefs in target physics [11].

## Materials and Methods

### Participants

26 right-handed observers (female: 17, mean age: 22, range: 18–31) took part in this study, which was conducted with local ethics approval at the Department of Psychiatry and Psychotherapy, Charité Universitätsmedizin Berlin, Germany. All participants had normal or corrected-to-normal vision and provided informed written consent to participate in this study.

### Exclusions

Due to technical problems, 7 out of 26 participants viewed the experiment at refresh rate of 75 Hz instead of 60 Hz, resulting in a slight overall increase in speed of presentation and consequently shorter block durations. For the sake of simplicity, we excluded these participants from analysis. Including these participants did not change the overall pattern of results. Given that one further participant had to be excluded for not following the experimental instructions correctly, the results presented here are based on 18 participants in total (female: 11, mean age: 22, range: 18–28).

### Stimulus and procedure

**Fig 1:**
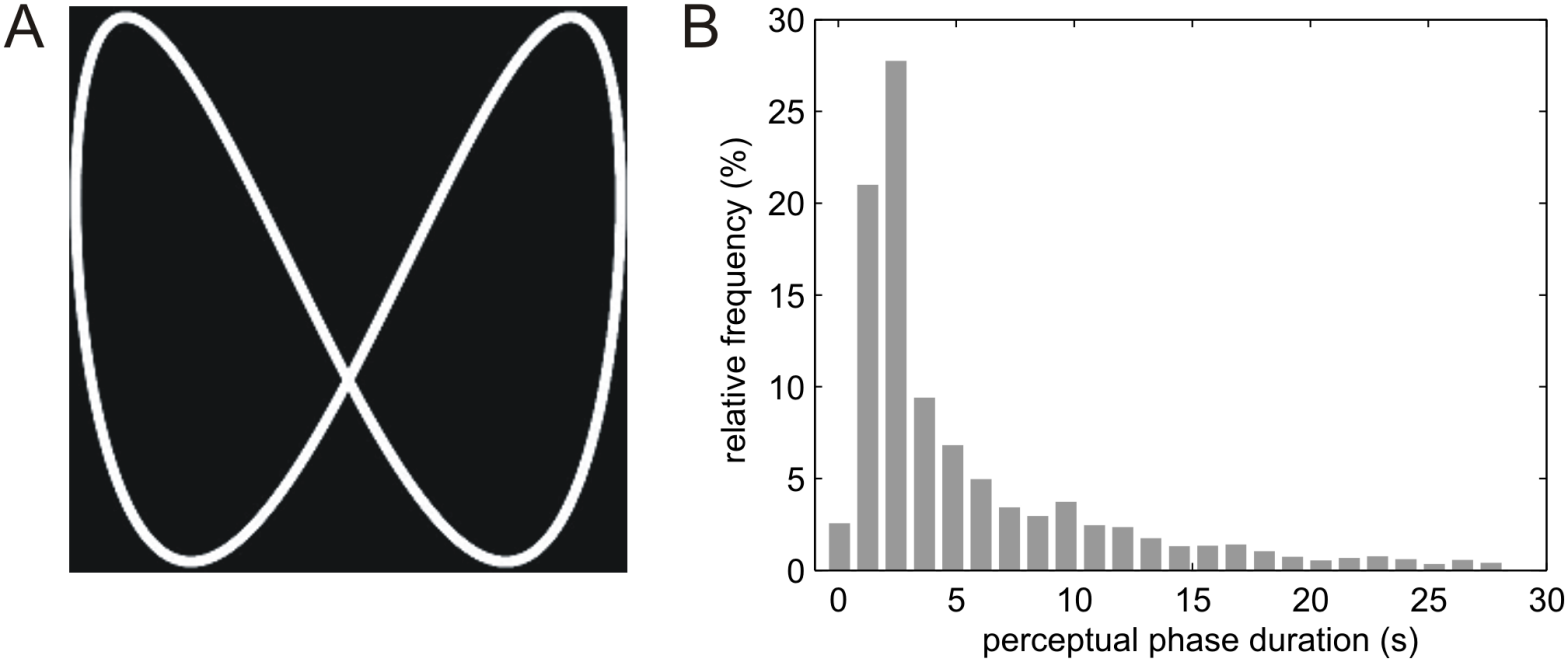
Lissajous figure and distribution of perceptual phase durations. (A) Lissajous are generated by the intersection of two sinusoids with perpendicular axes and increasing phase-shift whose frequency determines the speed of illusory 3D rotation. Following their introduction to experimental psychology, Lissajous figures were originally studied by means of twin-oscillators and analog cathode ray oscillographs in the 1940s and 1950s [6]. (B) Perceptual phase duration across all participants, runs and conditions. Distributions with a sharp rise and a long tail are typical of bistable perception.

Stimuli were generated using the Psychophysics Toolbox 3.0.9 [12] running under Matlab R2007b (Mathworks Inc., USA) and presented at 60 Hz for 19 participants, and at 75 Hz for the remaining 7 participants on a CRT monitor (SAMTRON 98 PDF, dimensions: 36.5 x 27.5cm, resolution: 1024 x 768 pixels) at a viewing distance of 60 cm. Participants observed moving Lissajous figures (Fig 1A), which were generated by the intersection of two sinusoids with perpendicular axes: *x*(*t*) = *sin*(*at*), *y*(*t*) = *cos*(*bt*+*δ*), with a = 3, b = 6, and *δ* increasing continuously from 0 to 2*π*.

The line width of the Lissajous figures was 0.10° visual angle. By presenting two identical figures separately to both eyes using a mirror stereoscope, we followed the experimental setup described by [9]. This enabled us to present a brief disambiguated training session at the beginning of the experiment, which we generated by introducing disparity cues (data not reported in results). We systematically varied shifting frequency and the size of the Lissajous figure in a 2 x 2 repeated measures design. The frequency at which the sinusoids were shifted against each other was set to 0.15 or 0.30 revolutions per second (rps). During one revolution, the sinusoids were shifted from 0 to 2*π*. Figure size was either 2.05° x 2.05° (250 x 250 pixels) or 4.10° x 4.10° (500 x 500 pixels).

Our two experimental manipulations had distinct effects on five parameters (a-d) of the Lissajous figure (summarized in Table 1): A twofold increase in shifting frequency leads to a twofold increase in the number of self-occlusion events (a), the planar-speed of the sinusoids (d) and speed of illusory rotation of the Lissajous figure (e) as well as a twofold reduction of the duration of single self-occlusion events (b). Please note the total duration of self-occlusion events (c = a x b) remains unchanged by the manipulation in shifting frequency. Conversely, a twofold increase in stimulus size results in a twofold reduction in the duration of single self-occlusion events (b) and the total duration of self-occlusion events (c) as well as a twofold increase in planar speed of the sinusoids. The number of self-occlusion events and the speed of illusory rotation remain unchanged by the manipulation in stimulus size. In total, our two-factorial design yielded 2 x 2 = 4 conditions.

During each run, which lasted 345 seconds, each condition was presented once in blocks of 80 seconds. Such blocks were separated by 5 seconds of fixation, and the order of conditions within each run was randomized. Participants completed 8–9 runs of the experiment, amounting to approximately 60 minutes of psychophysical testing per participant. Responses were recorded by a standard keyboard using the left arrow button for clock-wise (CW) and the right arrow button for counter-clock-wise (CCW) rotation of the Lissajous figure (viewed from top). The down arrow button was used to report unclear or mixed percepts. Participants were instructed to report their first perceived direction of rotation at stimulus onset with the first button press and indicate all further changes in perceived direction of rotation.

### Data analysis

To analyse the effect of shifting frequency and stimulus size on perceptual transitions of the Lissajous figure, we first performed a conventional analysis. Next, we performed a novel Bayesian analysis in the aim to estimate the precision at which a current percept influences on perceptual decisions at the consecutive self-occlusion configuration of the Lissajous figure.

#### Conventional analysis

In order to verify the common features of bistable Lissajous figures, we calculated the percentage of clear percepts (i.e., the number of CW and CCW responses divided by the total number of responses, including mixed percept responses), the distribution of perceptual phase durations across all conditions and participants and the distribution of button-presses relative to degrees of rotation of the Lissajous figure (ranging from 0° to 360°), i.e., to the phase shift of the two sinusoids (ranging from 0 to 2*π*). As our main dependent variable, we calculated mean transition probabilities, which reflect how fast percepts change [13]. Here, transition probabilities are defined by the number of perception transitions divided by the number of self-occluding events, thus denoting the probability that a self-occlusion is accompanied by a transition in perception. For every condition separately, these dependent variables were averaged first across runs and then across participants. Finally, the percentage of clear percepts and transition probabilities were submitted to repeated measures ANOVAs. We report partial eta squared (*ηp*^2^) as measure of effect size (SPSS 22.0 for Windows, IBM Corp. 2013).

#### Bayesian analysis

For Bayesian analysis, we designed a generative model of bistable perception based on the prediction of perceptual outcomes on a trial-by-trial basis. (The term ‘trial’ refers to the interval between two consecutive self-occluding configurations of the Lissajous figure: Given that perceptual transitions of the Lissajous figure occur almost exclusively at self-occluding configurations, we defined this as the sampling rate in our model and down-sampled the participants’ responses accordingly. Thus, every trial *t* corresponds to the interval between the self-occluding configurations t and t + 1).

This method frames perception as an inferential process in which perceptual decisions are based on posterior distributions. According to Bayes’ rule, such posterior distributions are derived from likelihood distributions representing the visual stimulation, and prior distributions reflecting a-priori knowledge about the visual world. Under Gaussian assumptions, the posterior distributions can be derived analytically [14]. Crucially, the impact of likelihood and prior on the posterior scales with their respective precision (i.e., the inverse variance).

**Fig 2:**
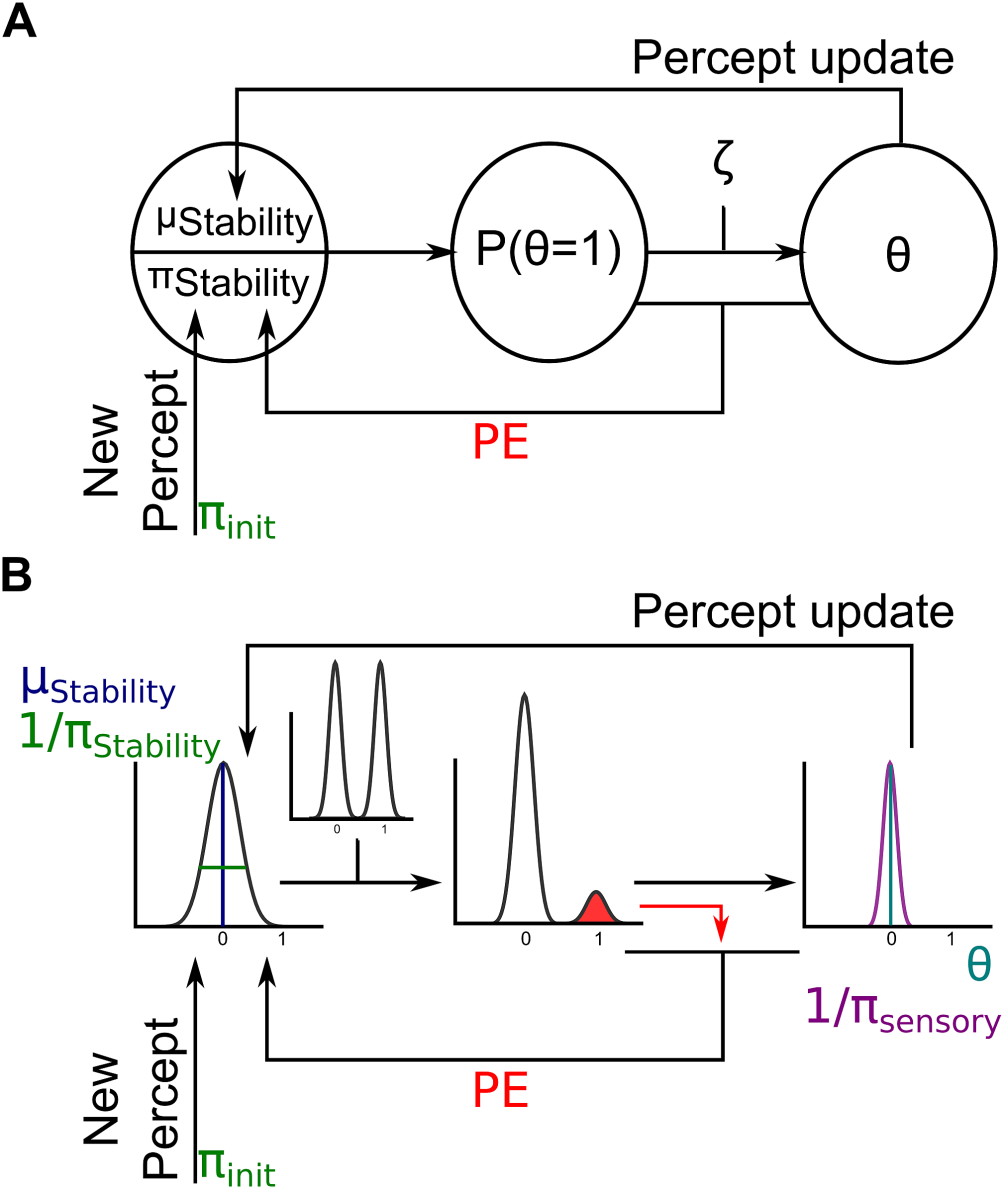
Bayesian modeling procedure. (A) The prior distribution ‘perceptual stability’ is defined by its mean *μ*_*stability*_ (corresponding to the perceptual decision made at the last self-occluding configuration, see ‘Percept update’-arrow) and its precision *π*_*stability*_. If a new percept was reported at the preceding trial, *π*_*stability*_ is set to *π*_*init*_ produce a weighted bimodal distribution *P*(*θ*), which is in a next step transformed by a unit-sigmoid function determined by parameter *ζ* and used to predict the perceptual outcome *θ*. The difference between *P*(*θ* = 1) and *θ* constitutes the prediction error (PE). (B) In this illustration with exemplary Gaussian probability distributions, the prior distribution ‘perceptual stability’ is defined by its mean *μ*_*stability*_ (blue line) and its precision *π*_*stability*_ (the inverse of its variance depicted in green). This prior distribution is combined with a bimodal likelihood distribution. The weighted bimodal distribution is used to predict the percept indicated by the subject at that trial (defined by its mean *θ* depicted in cyan and the inverse of its variance shown in purple). The difference between the weighted bimodal distribution and the percept is highlighted in red and constitutes a prediction error signal, which is used to adjust the prior distribution ‘perceptual stability’.

Bistable perception can be conceived to result from the sampling of a bimodal posterior distribution [15], which in turn originates from the combination of a bimodal likelihood with unimodal prior distributions. For the context of this experiment, we assume that the current percept constitutes a ‘perceptual stability’-prior for the visual system, the mean of which corresponds to the current percept, while its impact on visual perception is represented by its precision that we estimate for every condition separately. Importantly, we allow the prior precision to be affected by prediction error signals, which enables the modelling of a dynamic process eliciting transitions in perception. Furthermore, it allows for the estimation of the strength of the implicit prediction of perceptual stability (as represented by the precision of the prior distribution ‘perceptual stability’) and thus mirrors the idea of “representational momentum”. A schematic depiction of the modelling procedure—which we describe in detail below—can be found in Fig 2.

In this experiment, we presented moving Lissajous figures which could be perceived as rotating either CW or CCW. In our modelling analysis, we represent this stimulus by a bimodal likelihood distribution, while the two alternative percepts are given by:

$$\theta =\left\{\begin{array}{cc} 1: & \;\to \;(rotation)\\ 0: & \;\leftarrow \;(rotation) \end{array}\right.$$ (1)

The mean of the prior distribution ‘perceptual stability’ (*μ*_*stability*_) at trial *t* was defined by the percept indicated by the participant at the preceding trial:

$$\mu_{stability}\left(t\right)=\theta \left(t-1\right)$$ (2)

The precision of the prior distribution ‘perceptual stability’ (*π*_*stability*_) in trial *t* was defined as follows: If a new perceptual decision was made in trial *t* − 1 (i.e., *t* − 1 = *t*_0_), *π*_*stability*_ was set to an intial precision *π*_*init*_. In all other cases, *π*_*stability*_ was calculated by subtracting a precision-weighted prediction error (PE) from the precision at the preceding trial:

$$\pi_{stability}\left(t=t_{0}\right)=\pi_{init}$$ (3)

$$\pi_{stability}\left(t\neq t_{0}\right)=\pi_{stability}\left(t-1\right)*exp(-\frac{\pi_{sensory}}{\pi_{stability}\left(t-1\right)}|PE\left(t-1\right)\left|\right)$$ (4)

The precision-weight is given by the fraction between the precision of the sensory representation *π*_*sensory*_ and the current *π*_*stability*_.

We derived a posterior probability of CW rotation *P*(*θ* = 1) by weighting the bimodal likelihood distribution with the prior distribution *π*_*stability*_ [15]:

$$r=\frac{P(\theta =0)}{P(\theta =1)}=exp\left(\frac{{\left(\theta_{0}-\mu_{stability}\right)}^{2}-{\left(\theta_{1}-\mu_{stability}\right)}^{2}}{\pi_{stability}^{-2}}\right)$$ (5)

$$P\left(\theta =1\right)=\frac{1}{r+1}$$ (6)

In order to predict participants’ responses *θ*(*t*), we applied a unit sigmoid function to *P*(*θ* = 1):

$$\theta \left(t\right)=\frac{P{\left(\theta =1\right)}^{\zeta }}{P{\left(\theta =1\right)}^{\zeta }+{\left(1-P\left(\theta =1\right)\right)}^{\zeta }}$$ (7)

The prediction error was calculated by subtracting *P*(*θ* = 1) from actual percept indicated by the participants’ *θ*:

PE(t)=θ(t)-P(θ=1)(t) (8)

We used Bayesian model inversion to estimate the precision of the initial precision of the prior distribution ‘perceptual stability’ (*π*_*init*_) separately for all conditions. This method maximises the log-model evidence by minimising the surprise about the data of individual participants as approximated by negative free energy [16]. For model inversion, precisions were modelled as log-normal distributions. *π*_*init*_ was estimated as a free parameter for the four conditions separately, each of which had a prior mean of log(1) and a prior variance of 1. All other parameters were fixed (i.e., a prior variance of 0) and set to *π*_*sensory*_ = 1 and *ζ* = 1 in both models. We compared this model (model A) to a control model (model B) in which *π*_*init*_ and *π*_*sensory*_ were set fixed to zero and thus effectively removed from the model. Parameters were optimised using the quasi-Newton Broyden-Fletcher-Goldfarb-Shanno minimisation algorithm as implemented in the HGF 4.0 toolbox (distributed as part of the TAPAS toolbox, http://www.translationalneuromodeling.org/tapas/). For model level inference, we calculated exceedance probabilities (i.e., the probability that model A is better at explaining the observed data than model B) using random effects Bayesian model selection [17] as implemented in SPM12 (http://www.fil.ion.ucl.ac.uk/spm/software/spm12/). From the winning model, we report posterior parameter estimates for *π*_*init*_ separately for each condition, averaged first across runs and then across participants.

## Results

### Conventional analysis

On average, the middle button indicating mixed or otherwise unclear percepts was rarely used (4% of all button presses), indicating that participants had a clear impression of the illusory rotation in the vast majority of cases throughout the experiment. In all conditions, the mean percentage of clear percepts was above 94%, and it was not significantly modulated by our experimental factors (shifting frequency: F(1,17) = 2.51, p = .131, *ηp*^2^ = .13; size: F(1,17) = .10, p = .762, *ηp*^2^ = .01; interaction: F(1,17) = .73, p = .406, *ηp*^2^ = .04).

**Fig 3:**
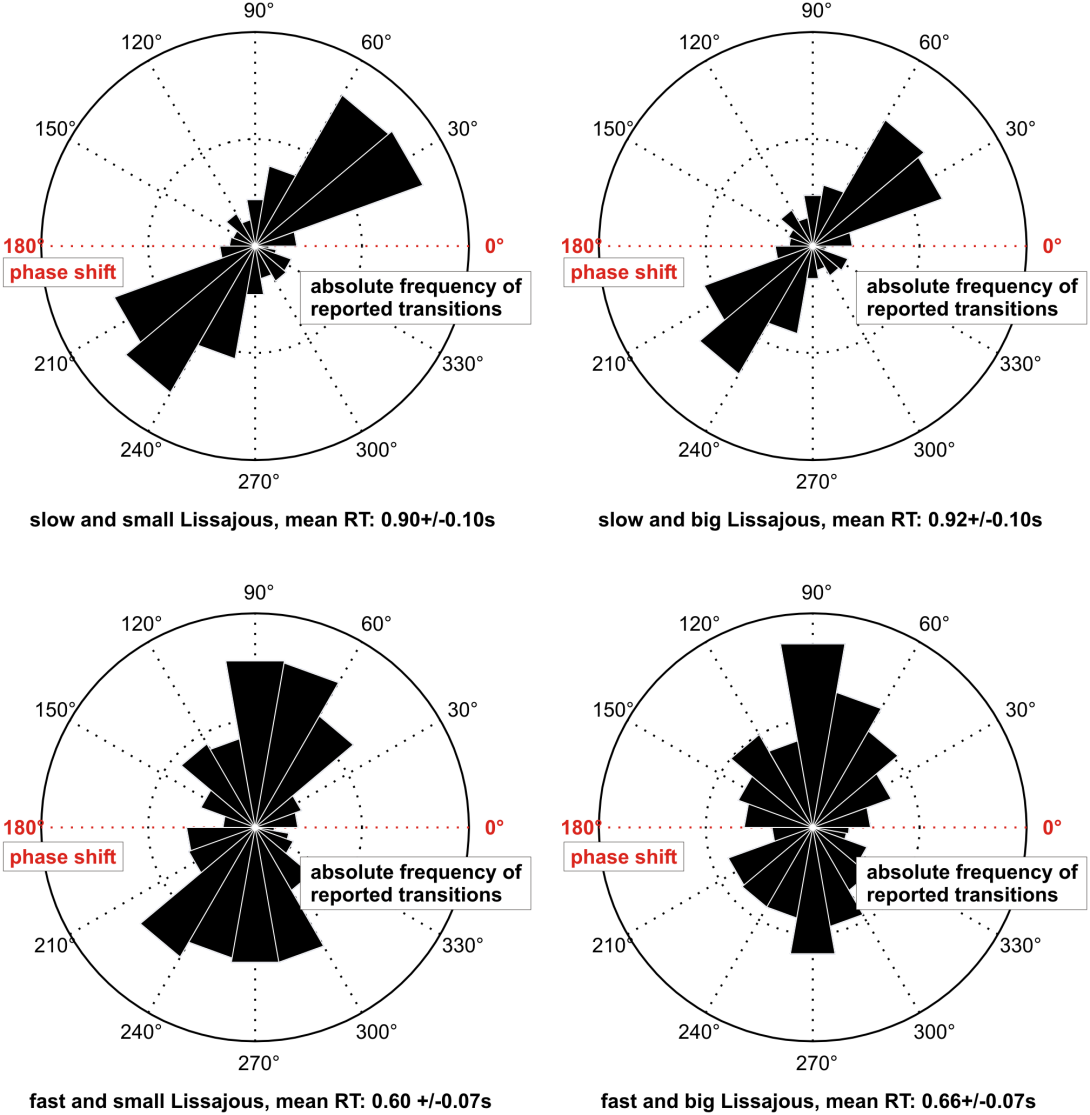
Distribution of button presses indicating perceptual transitions. Separately for all four conditions, transitions are plotted relative to the phase shift (i.e., degree of illusory rotation, 0–360°) of the Lissajous figure. The four panels show absolute frequencies of reported transitions across all participants. For the “slow” conditions (upper panels), the inner dashed circles of the polar plots denote 100, the outer circles denote 200. For the “fast” conditions (lower panels), the inner dashed circles of the polar plots denote 50, the outer circles denote 100. Self-occlusions of the Lissajous figure occurred at 0 and 180° (indicated by red color). Note that 10° of illusory rotation correspond to 0.2s in the “slow” and 0.1s in the “fast” conditions, leading to an increased offset between the self-occlusion positions and the distribution of transitions relative to degree of illusory rotation. Response times (RT) in all four conditions were calculated as the difference between the time points of button presses and the onsets of the preceding overlaps and are expressed as mean ± standard error of the mean.

Fig 1B shows that across participants and conditions, the distribution of perceptual phase durations was unimodal and followed a sharp rise and a slow fall. The time point of button presses relative to degrees of rotation of the Lissajous figure (ranging from 0° to 360°), i.e., to the phase shift of the two sinusoids, followed a bimodal distribution, reflecting the existence of two critical configurations of the Lissajous figure facilitating perceptual switches (Fig 3). As it has been described in a number of previous studies [7–9], these critical configurations relate to the self-occlusion events (at 0° and 180°) of the Lissajous figure.

**Fig 4:**
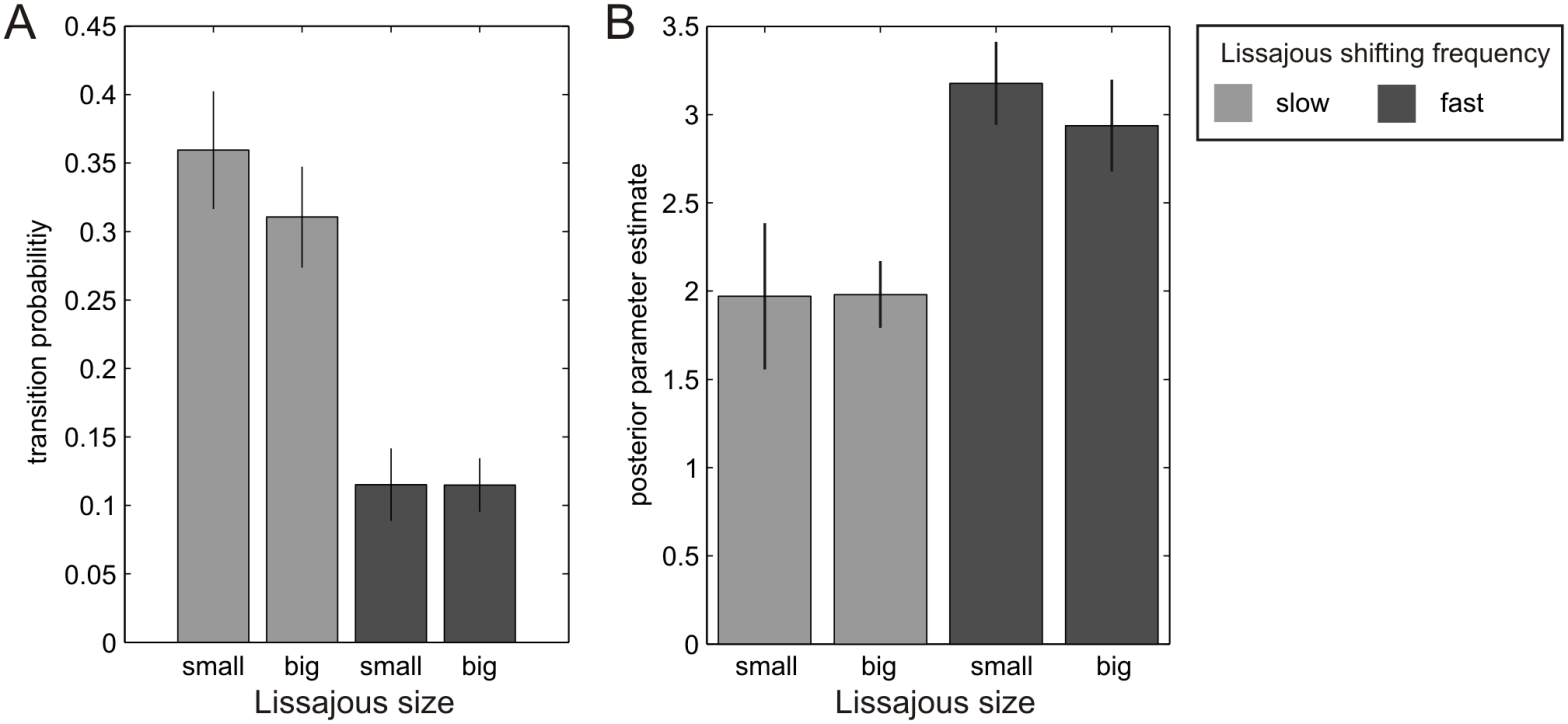
Results of the standard and Bayesian data analysis. (A) Average transition probabilities separately for the four conditions, averaged across runs and participants. Error bars indicate the standard error of the mean. (B) Posterior parameter estimates for *π*_*init*_. Posterior parameters averaged across runs and participants, separately for all conditions.

Fig 4A shows mean transition probabilities for all four experimental conditions. Lissajous figures rotating of high shifting frequency showed lower transition probabilities than slower stimuli (0.12 vs.0.34), and the main effect of shifting frequency was significant (F(1,17) = 66.49, p < .001, *ηp*^2^ = .80). Again, we found no significant effect of stimulus size (F(1,17) = 1.17, p = .294, *ηp*^2^ = .06). Smaller stimuli elicited transition probabilities of 0.24, while bigger Lissajous figures induced transitions at a probability of 0.21 at self-occluding configurations. The interaction between both factors was not significant (F(1,17) = 2.57, p = .127, *ηp*^2^ = .13). Please note that when basing the analysis on phase durations instead of transitions probabilities, the results of our summary statistics remained qualitatively identical.

### Bayesian analysis

On a model level, we compared a model that allowed for the estimation of a prior distribution ‘perceptual stability’ (model A) to a control model which did not incorporate such a prior (model B). Random effects BMS (Stephan et al., 2009) indicated an exceedance probability of above 99% for model A. Within this model, we calculated the mean of the posterior parameter estimates for the initial precision of the prior distribution ‘perceptual stability’ (*π*_*init*_), separately for all experimental conditions (Fig 4B). Here, stimuli eliciting higher speeds of illusory rotation yielded higher posterior parameters estimates for *π*_*init*_ as compared to their slower counterparts (3.06 vs. 1.98). We found a significant main effect of ‘shifting frequency’ (F(1,17) = 10.11, p = .005, *ηp*^2^ = .37). In further agreement with the conventional analyses, we did not observe a significant main effect of stimulus size (F(1,17) = .26, p = .619, *ηp*^2^ = .02): Here, the posterior estimate for *π*_*init*_ was 2.57 for smaller and 2.46 for bigger stimuli. The interaction between both factors was not significant (F(1,17) = .43, p = .520, *ηp*^2^ = .03).

## Discussion

The first goal of our study was to disentangle two hypotheses regarding the finding of lower transition probabilities for Lissajous figures at higher speed of illusory of rotation. According to one hypothesis, the duration of self-occlusions determines the perceptual stability of the Lissajous figure. Alternatively, we hypothesized that increased illusory rotational speed stabilizes the perception of the Lissajous figure due to “representational momentum”. We manipulated the shifting frequency and size of the Lissajous stimulus and tested the effects of these manipulations on transition probabilities.

In the conventional analysis, we found a main effect of shifting frequency, but no effect of size on Lissajous transition probabilities. Therefore, we conclude that our data support the notion that “representational momentum” is associated with the perception of the ambiguous Lissajous figure. Originally, this term referred to the forward displacement that is observed when participants have to judge the final position of a previously viewed target and often deviate from the true target position in the direction of target motion [18]. With linear physical momentum being equivalent to the product of an object’s mass and velocity, common theories of representational momentum focussed on the incorporation of physical properties in mental representations [18], or on implicit beliefs about the physics of the target stimulus [19]. For instance, stronger displacement has been observed for higher target speeds [20] and accelerations [18]. Interestingly, size or inferred mass of the object associated with the stimulus did not seem to result in different judgements on final target position [21]. This would be in line with our observation that larger stimulus size had no significant effect on the perceptual dynamics of the Lissajous figure. Beyond the similarity between studies, the absence of a size effect clearly marks one of the limitations of the perceptual momentum account: As angular momentum is formally defined by the product of the mass of the body, the radius of the orbit, and the square of the angular velocity, one would also expect an effect of stimulus size.

One might argue that the observed perceptual stabilization at higher rotational speed was not due to a true change in perceptual dynamics, but rather due to the limited ability of participants to report these dynamics. It is well known that a rapid succession of perceptual events demanding behavioral report can result in a psychological refractory period, or PRP, at the level of response selection [22]. However, we consider the PRP hypothesis unlikely in our case because the fast condition still gave participants ample time to respond: at 0.30 rps, there were 0.60 critical stimulus configurations (i.e., self-occlusions) per second, and therefore the minimal interval between two self-occlusions was 1.67s.

Crucially, for a given Lissajous figure of constant complexity, the speed of illusory rotation cannot be changed without changing the number of self-occlusion events that occur during a fixed time interval. In a previous study [9], we assessed the effect of an increased number of self-occlusion events per time interval by increasing the complexity of the Lissajous figure. In contrast to our present results, this earlier study showed a reduction of perceptual stability when the number of self-occlusion events increased. This previous finding thus speaks against the interpretation that it might have been the higher number of self-occlusion events per time interval that caused the increased perceptual stability at higher rotational speed observed in our present study.

The data presented here does not show a main effect of overlap duration (as manipulated by stimulus size), which is in contrast to our previous work, in which overlap duration (as manipulated by Lissajous line-thickness) did lead to a significant decrease in perceptual stability of the Lissajous figure. One plausible reason for this might be that our manipulation of stimulus size lead to substantially smaller changes in overlap duration (two-fold) as compared to our previous manipulation of line-thickness (six-fold).

Interestingly, a recent study [23] reported decreased perceptual stability for structure from motion spheres characterized by lower dot density and higher rotational frequency. The latter finding stands in contrast to increased perceptual stability found for Lissajous figures rotating at higher speeds, which might be due to the existence of depth-symmetric stimulus configurations in Lissajous stimuli. To further investigate this intriguing discrepancy, one could study transition probabilities for different rotational speeds while parametrically varying the depth-symmetry of the stimulus.

The common ground for representational momentum and the effect of rotational speed described here might be that both are conveyed by anticipation, or from a predictive coding perspective, “predictions” or “beliefs” [24]. Such internal predictions about the presented stimulus (e.g., the learned physical properties of real-world objects) could act as prior distributions on stimulus representations and change the contents of perception. Corresponding with this intuition, expectations regarding changes in target motion were indeed shown to affect perceptual judgements about the final target position [25]. As to how such anticipations, predictions or beliefs might emerge, one can speculate that they mirror regularities in the environment that shape our predictions through learning. Along this line of argument, the observation that real world objects rotating at higher speeds are less likely to change their direction of rotation may foster a prediction that manifests itself by its influence on perception, most markedly under ambiguous viewing conditions.

It is noteworthy that in contrast to classical experiments on representational momentum, the perceived speed of rotation of the Lissajous figure cannot be directly related the physical properties of the stimulus itself (since it seems not related to planar pixel velocity), but emerges on the ground of the illusory percept. However, if one assumes that the illusory percept is the result of an inferential process that is, in principle, equal for all types of presented stimuli and leads to a perceptual experience closely resembling perception under unambiguous conditions [7], it is not surprising that effects such as “representational momentum” can be evoked by ambiguous and unambiguous stimuli alike.

The second goal of our experiment was to formulate the effect of increased stability of the Lissajous figure within the predictive coding framework. Specifically, we aimed at testing whether differences in the features of the presented stimuli would correspond to differences in the strength (or precision) of predictions that influence the time course of bistable perception, thereby quantifying the influence of Lissajous shifting frequency and size on the stability of the visual environment in a trial-by-trial fashion.

Within our winning model, parameter level inference indicated a higher posterior initial precision for the prior distribution ‘perceptual stability’ for stimuli of higher shifting frequency as compared to stimuli of lower shifting frequency, whereas we did not find a significant difference between different Lissajous sizes. The prior distribution ‘perceptual stability’ can be viewed in analogy to an implicit prediction of perceptual stability, which we found to be increased for Lissajous figures rotating at higher speeds, nicely mirroring the results from the conventional analysis.

By framing bistable perception as an inferential process resting on the computation of posterior distributions from prior and likelihood, we can model changes in the perceptual stability of a stimulus as the result of differences in the precision of predictions. This perspective on bistable perception is thus opposed to the classical dichotomy between ambiguous and unambiguous visual environments. We argue that under normal everyday viewing conditions, the visual scene offers a number of contextual cues (such as binocular disparity, shading, etc.) that act as priors for perception and effectively reduce the “bistable potential” evoked by the constantly ambiguous sensory input that we receive through our retinae, thereby yielding a clear and stable experience of our visual environment. We propose that it is the lack of such contextual cues or priors that lead to a breakdown of perceptual stability characterizing bistable perception.

Crucially, predictive coding models of bistable perception can be used to test whether a given factor such as representational momentum constitutes a contextual cue aiding in the disambiguation of the presented stimulus and to quantify its impact on perception. The method introduced here can therefore be used to compare the impact of prior distributions on perceptual stability between individual participants, and constitutes a flexible tool to study the mechanisms that construct a stable impression of our environment despite inherently ambiguous sensory inputs. Beyond corroborating the results of conventional approaches, generative predictive coding models thus provide a new perspective on bistable perception, which focuses on the interplay between implicit prior predictions and prediction errors elicited by the ambiguous sensory stimulation. Besides providing a different theoretical point of view, they might also be applicable for the investigation of the neural correlates of perceptual transitions in ambiguity. Ultimately, this approach to bistable perception could, for example, constitute an effective tool to study perceptual alterations in neuropsychiatric disorders such as schizophrenia [26], for which experiments on representational momentum have shown greater displacement in direction of target motion [27].
